# Essential Oil from *Coriandrum sativum*: A review on Its Phytochemistry and Biological Activity

**DOI:** 10.3390/molecules28020696

**Published:** 2023-01-10

**Authors:** Jameel M Al-Khayri, Akshatha Banadka, Murali Nandhini, Praveen Nagella, Muneera Q. Al-Mssallem, Fatima M. Alessa

**Affiliations:** 1 Department of Agricultural Biotechnology, College of Agriculture and Food Sciences, King Faisal University, Al-Ahsa 31982, Saudi Arabia; 2 Department of Life Sciences, CHRIST (Deemed to Be University), Bangalore 560029, India; 3 Department of Life Sciences, School of Sciences, JAIN (Deemed to Be University), Bangalore 560027, India; 4 Department of Food Science and Nutrition, College of Agriculture and Food Sciences, King Faisal University, Al-Ahsa 31982, Saudi Arabia

**Keywords:** coriander essential oil (CEO), phytochemistry, EO variation, biological activity, antimicrobial

## Abstract

Essential oils are hydrophobic liquids produced as secondary metabolites by specialized secretory tissues in the leaves, seeds, flowers, bark and wood of the plant, and they play an important ecological role in plants. Essential oils have been used in various traditional healing systems due to their pharmaceutical properties, and are reported to be a suitable replacement for chemical and synthetic drugs that come with adverse side effects. Thus, currently, various plant sources for essential oil production have been explored. Coriander essential oil, obtained from the leaf and seed oil of *Coriandrum sativum*, has been reported to have various biological activities. Apart from its application in food preservation, the oil has many pharmacological properties, including allelopathic properties. The present review discusses the phytochemical composition of the seed and leaf oil of coriander and the variation of the essential oil across various germplasms, accessions, at different growth stages and across various regions. Furthermore, the study explores various extraction and quantification methods for coriander essential oils. The study also provides detailed information on various pharmacological properties of essential oils, such as antimicrobial, anthelmintic, insecticidal, allelopathic, antioxidant, antidiabetic, anticonvulsive, antidepressant, and hepatoprotective properties, as well as playing a major role in maintaining good digestive health. Coriander essential oil is one of the most promising alternatives in the food and pharmaceutical industries.

## 1. Introduction

Essential oil (EO) is a hydrophobic liquid product of secondary metabolism in plants that contains volatile compounds [1]. From the dawn of history, this valuable plant product has been extensively used in medicine [2]. It is also used as a flavoring agent and for food preservation. Theophrastus Bombastus von Hohenheim, often known as Paracelsus, a Swiss physician, coined the term “essential oil” in the fifteenth century from the word “quinta essentia”, an active ingredient of a drug preparation [3]. EO is also known by other names, such as aetheroleum, ethereal oils, and volatile oils [4]. EOs contain hundreds of potential chemical compounds with a low molecular weight ranging from 50–200 Da in various combinations [5]. According to their chemical structures, these organic chemical compounds are categorized into four groups: terpenes (mono- and sesquiterpene), terpenoids (alcohols, aldehydes, epoxides, esters, ethers, ketones, and phenols), phenylpropenes, and sulfur- and nitrogen-derived aromatic compounds [6].

EOs are produced by different biosynthetic pathways in various species as a defense mechanism in response to various biotic and abiotic stressors. The functional importance of EO is not just restricted to the plant but also benefits humans [7]. EOs show biological properties such as anticancer [8], anti-inflammatory, antioxidant [9], and antimicrobial [10]. Due to their wide range of applications in the food and pharmaceutical industries, there is a huge demand for essential oil production on a large scale. Furthermore, the plants are evaluated for their ability to produce essential oil. EO production varies greatly in different plants based on the environment and the growth conditions. Cedar wood, cinnamon, citronella, clove, eucalyptus, lavender, lemongrass and rosemary are a few essential oils that have been traditionally used by people for a variety of purposes around the globe [11]. *Coriandrum sativum* L. is one such high EO bearing medicinal plant that contains EO in its leaves, stems, flowers, fruits, and seeds [2].

*Coriandrum sativum* L. (*Coriandrum diversifolium* Gilib., *Coriandrum majus* Gouan, *Coriandrum testiculatum* Lour., *Selinum Coriandrum* (Vest) E. H. L. Krause) is a culinary herb that belongs to the family Apiaceae (Umbelliferae). It is referred to as Kusthumbari (Sanskrit), Dhania (Hindi), Kottamalli (Tamil), Kothimeera (Telugu), Kottambari (Kannada) and many other names [12]. At present, coriander has been cultivated globally in countries such as the USA, the UK, Argentina, India, France, Italy, Morocco, Myanmar, Mexico, Netherlands, Pakistan, Turkey, Spain, and Romania [13]. Coriander is easily identifiable due to its unique morphology. This erect annual herb has a predominant taproot system and thin, soft branching stems, with a height of 20–70 cm [2]. The pale/dark green leaves are glabrous, lanceolate, lobed, and variable in shape. The white or light pink flowers are first borne in peripheral umbellets of small umbels. The peripheral flowers are protandrous and the central flowers are staminiferous or sometimes sterile [12]. The seeds of the plants appear almost ovate and globular. It is a dry schizocarp with multiple longitudinal ridges on the surface and two mericarps [14].

*Coriandrum sativum* is a tropical crop that thrives best in a frost-free, relatively dry tropical and subtropical climate. It is a commercial crop that can be found growing in a variety of habitats, including gardens, open spaces, plains, hills, and railroads (Datiles, 2020). It grows well in a climate with an average rainfall of 75–100 mm and a temperature between 15–28 ℃. The plant prefers to grow in loamy soil that drains well with a soil pH of 8–10. The best season to grow coriander is summer in temperate zones and cooler season in subtropical/tropical zones [15].

Although the entire *C. sativum* plant is edible, the fresh leaves and dried seeds are the parts that are most frequently used [16]. Its green foliage, which is rich in proteins (33 mg/g), vitamins (0.1 mg/g), minerals such as calcium (1.4 mg/g), phosphorus (0.6 mg/g), and iron (0.1 mg/g), and carbohydrates (65 mg/g), is used as a vegetable and in salads [17]. This citrus-flavored spice is widely used for cooking purposes. Coriander seeds are employed in pickling and brining processes. Stews, soups, and salads can all contain the spice of ground coriander seeds. The leaves are used as salad toppings, garnishes, and as in flavoring beverages and mayonnaise. In addition to being blended into rich sauces, whole coriander is used in spice rubs [16]. Apart from its uses in cooking, coriander is reported to contain secondary metabolites that account for its medicinal properties such as anticonvulsant, antidiabetic, antimicrobial, antioxidant, etc. [18].

One of the characteristic features of *C. sativum* is its ability to produce essential oil. Coriander essential oil is a triglyceride oil containing a monounsaturated fatty acid called petroselinic acid. The essential oil is extracted mostly from the seeds by various extraction methods such as hydrodistillation, soxhlet and supercritical CO_2_ extraction methods. It is also extracted from leaves, stems, roots, bark, and flowers, and the EO content and composition vary in different parts of the plant [2]. Borneol, cineole, coriandrol, cymene, dipentene, geraniol, linalool, phellandrene terpineol, and terpinolene are the principal components of the EO [19]. The essential oil pressed from the seeds appears as a pale yellow or colorless liquid with a distinctive odor and a warm, sweet, mild aromatic flavor [2]. Coriander seed oil has a density of 0.831 g/cm^3^ and a refractive index of 1.4592 [20]. This underrated oil has great advantages. It gives food products their characteristic flavor and serves as a preservative by inhibiting the lipid peroxidation of processed meat products.

The phytochemicals present in the oil such as linalool, a volatile flavor compound, confer medicinal properties. Essential oil uses are most prevalent in relation to the digestive system. The essential oil offers excellent mental relief, particularly when used in aromatherapy, and is also effective for treating muscular issues such as aches and spasms [21]. Due to the growing demand for enhanced production of pharmacologically important essential oils, this study identifies coriander as an efficient EO bearer and emphasizes the phytochemical composition, extraction, and quantification methods of essential oils from *C. sativum*. Furthermore, the study discusses the various studies conducted on applications of essential oils derived from *C. sativum* in food and medicine.

## 2. Phytochemistry of CEO

The coriander plant harbors a wide range of phytochemicals which comprises essential oil, polyphenols, fatty acids, tocol, sterol, carotenoids, etc. The essential oil can be extracted from leaves, stems, flowers, pericarp, seeds and roots. The yield of essential oil from coriander seeds has been found to be in the range of 0.18 to 1.40% (*v*/*w*) [22]. The composition of the EO varies depending on the part of the plant, picking time, genotypes, environmental conditions, etc. [23]. Generally, the constituents of essential oil are divided into four different categories, namely terpenes, terpenoids, straight chain compounds, phenylpropanoids, and sulfur- or nitrogen-containing compounds [24]. It is reported by BACIS [25] that the essential oil of coriander contains more than 200 constituents, with 18 major components contributing to 97% of the total oil composition.

Essential oil of coriander comprises 60–80% terpene alcohols (linalool), 30% terpenes, ketones, esters and aromatic acids (Table 1). The trace constituents are coumarins/furanocoumarins: umbelliferone and bergapten. Linalool (2,6-dimethyl-2,7-octadien-6-ol) is a monoterpene compound, commonly found as a major component of coriander essential oil. It has been found to have numerous biological properties including antimicrobial, anticancer, anti-inflammatory, antidepressant, neuroprotective, hepatoprotective and lung protective activity. The mechanism of linalool showing anticancer activity is through the induction of apoptosis in cancer cells through oxidative stress and antimicrobial activity by disruption of the cell membranes of microbes [26].

### Coriander Seed Oil and Leaf Oil

**Figure 1:**
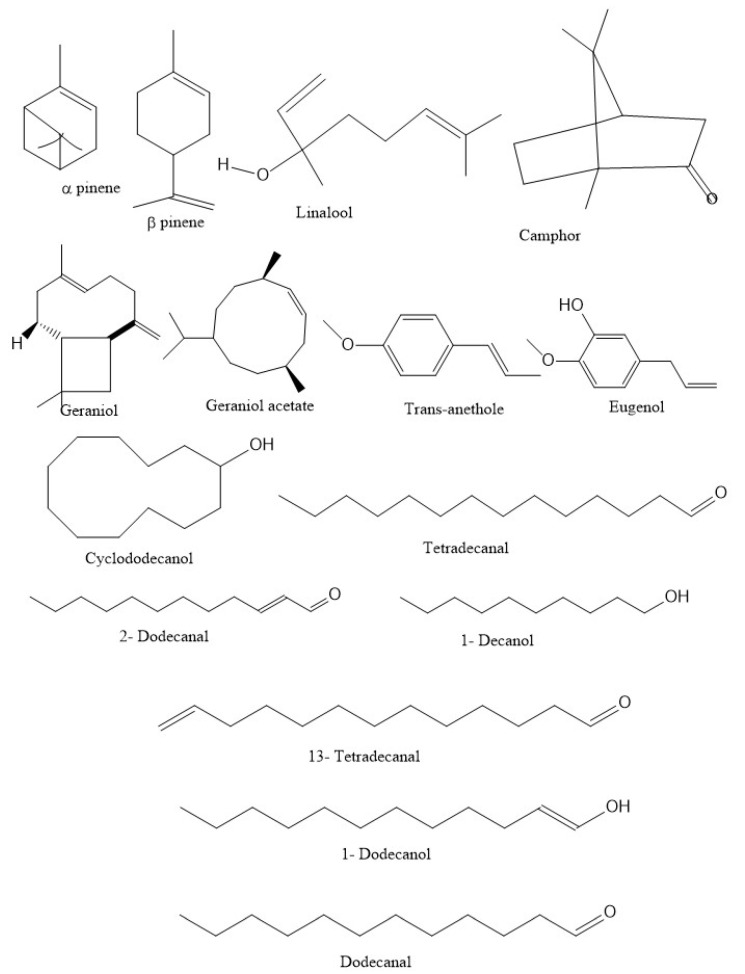
Chemical structures of some major constituents of coriander leaf and seed oil.

The oil from dried seeds is a pale yellow or colorless liquid with a distinctive pleasant odor and a mild, sweet, warm and aromatic flavor. The leaf oil has an unpleasant odor due to the presence of aliphatic aldehydes. The chemical composition of leaf oil and seed oil are given in Table 2. Bhuiyan et al. [28] have reported the different constituents of leaf and seed oil, where they have enumerated 53 different constituents from seed oil and 44 different compounds from leaf oil. Some of the important chemical constituents from seed and leaf oil are presented in Figure 1. Chung et al. [29] have extracted EO from leaf and stem and found that the EO content was 0.45 and 0.20%, respectively. The major components of stem were phytol (61.86%), 15-methyltricyclo[6.5.2(13,14),0(7,15)]- pentadeca-1,3,5,7,9,11,13-heptene (7.01%), dodecanal (3.18%) and 1-dodecanol (2.47%).

## 3. Variation in EO across Varieties/Germplasm/Accessions

The changes in soil and climatic conditions, altitude, irrigation, and other biotic and abiotic factors affect the EO composition. These factors have led to the evolution of different variants and chemotypes with slight variations in EO composition [58]. The major variations are the increase/decrease in oil yield, linalool content and presence of trace components (Table 3). Nejad et al. has analyzed 19 accessions of coriander in Iran and found an astounding variation in the oil yield ranging from 0.1% to 0.36%, linalool content 60–70%, terpinene 5.8–13.6%, pinene 2.8–7.1% and *p*-cymene 1.1–3.6% [58]. All the accessions were reported to contain 34 different constituents. In the study conducted by Zheljazkov et al. [59] the coriander seeds grown in the cool, wet conditions of Atlantic Canada yielded a higher oil content. Cultivars ‘Jantar’ and ‘Alekseevski’ were tested at an earlier seeding date for two years and it was observed that they contained high essential oil content.

It is reported that infraspecific classification of coriander can be done based on the quantity of linalool, camphor, limonene, and myrcene present in the seed oil [12]. Lopez et al. [63] and Saxena et al. [64] have analyzed 60 and 148 germplasms of coriander and have grouped them into five and ten groups, respectively, based on the composition of the EO. The cluster analysis or grouping of varieties based on the EO content allows the commercialization of coriander for various applications, as different purposes require different compositions of oil.

## 4. Variation of Essential Oil at Different Stages

The composition of EO slightly increases or decreases at different stages of growth due to changes in the chemical composition during the plant growth [65,66,67]. Fatty acid composition and oil content was also found to vary according to the maturity of seeds [68]. The EO content in coriander varies at different stages of its development. EO was tested at three stages of development of coriander by Msaada et al. (2007) and it was found that geranyl acetate, linalool, nerol and neral were found in immature fruits. Linalool, cis-dihydrocarvone and geranyl acetate were reported in the middle stage of development. Mature fruits consisted of linalool and cis-dihydrocarvone [69]. Telci et al. [70] have reported that the unripe fruits contain high EO content, with especially linalool content being higher in unripe fruits. It is observed that monounsaturated fatty acids increase and saturated polyunsaturated fatty acids decrease in the later developmental stages. The plants in earlier stages of development are highly nutritious, whereas the later stages were found to be important for industrial applications [71].

## 5. Variation of Essential Oil Constituents from Different Geographical Locations

The same variety of coriander cultivated in different geographical regions will have significant changes in the composition of EO depending upon the climatic conditions, seasons, phenological stages, method of extraction etc. The variations have been supported by various authors from different regions of the world [72,73]. Meteorological conditions like weather, temperature, rainfall between years of field studies also affected the EO content [74]. Linalool content of coriander grown in different ecological conditions showed a significant variation, i.e., a European variety yielded 73.1% and an Argentinean variety yielded 82.9% [74,75]. Fatty acid profiles, EO content, etc., varied significantly among the different coriander growing regions and stages of maturity. The main components linalool and geranyl acetate were found to vary with different geographical locations (Table 4). Growing regions and their interactions have a strong effect on 35 compounds. α-pinene, limonene, sabinene, cis-dihydrocarvone, γ-terpinene and geranial were not affected by change in regional factors [76]. The changes in composition according to geographical locations create a wide combination of EO content for commercialization purposes. For selection of the different combinations of EO content, a thorough knowledge and research on coriander varieties grown in different ecological conditions and EO contents are mandatory. Thus, the study of EO content from different varieties, germplasms and geographical locations are significant.

## 6. Variation in EO Due to Other Factors

Apart from the abiotic factors, changes in agronomic factors also affect the yield of the essential oil content. A study conducted by Georgieva et al., [84]. using different foliar fertilizers such as Fertigrain, Poly Plant, and Masterblend, and confirmed the changes in the seed oil content from 2.9% to 9.6% due to the application of Masterblend. The composition of EO is also influenced by the amount of organic and inorganic fertilizers applied, and the macro and micronutrients supplied to the plant. There was a slight variation of 1–2% increase in the EO composition when the plant was supplemented with Nitrogen and Phosphorous (NP) and nutrients Fe, Mg, Mn, Zn, Cu, Mo, Ni and Co. [85]. Özyazici, 2021 [86] identified different fertilizer sources that also affected the EO content from 0.29 to 0.33% in the Erbaa variety of coriander. The same observation was given by Rasouli et al. [87] where the different fertilizer type affected the EO content from 30–37% when compared to the control.

## 7. Extraction and Analysis of Coriander EO

EO composition of plants is influenced by the extraction process. Extraction of EO is carried out in the main by a classical approach ie. hydrodistillation or steam distillation which includes boiling for 180 min. Hydrodistillation (HYD) and steam distillation (SD) results in hydrolysis and thermal degradation of compounds, thus, modern techniques are employed to minimize the degradation of compounds. Modern approaches of extraction include microwave assisted hydrodistillation (MAHD), soxhlet-dynamic headspace, ultrasound-assisted extraction, rapid solid–liquid dynamic extraction, soxhlet extraction using dichloromethane (30 min), supercritical CO_2_ extraction, and supercritical fluid extraction (200–300 bar at 35 °C, CO_2_—14 g/min). The analysis of the extracted EO is done by gas chromatography with helium as a carrier gas (250–300 °C, run time 30 min and injection volume of 0.1–0.5 μL) with flame ionization detector, and the mass is analyzed by mass spectrophotometer. Msaada et al. [88] performed a detailed comparison on different extraction methods of EO of coriander and found out that supercritical CO_2_ extraction offered the maximum yield of oil (5.8%) and minimum non-volatile compound contamination in EO. Further, the SC-CO_2_ extraction revealed its high efficiency and integrity of oil volatiles. MAHD extraction yield of EO was lesser (0.1%) in comparison to hydrodistillation (0.2%) [89]. The supercritical fluid extraction method allows for the manipulation of parameters such as temperature and pressure, which leads to the extraction of different components [90]. Pavlić, et al. [91] report that the soxhlet extraction yielded a higher percentage of camphor, geranyl-acetate, terpinene and a lower percentage of geraniol and linalool compared to steam distillation.

The above-mentioned methods have their own advantages and disadvantages which include operating cost, yield, quality and quantity of the extracts, and the apt method for extraction is chosen according to the target constituents to be extracted, target yield, etc. (Table 5). The yield of linalool was less in subcritical water extraction when subjected to higher temperatures (170 °C) because linalool converted into linalool oxide. Various studies have been conducted to optimize extraction conditions to obtain the maximum oil yield [92].

## 8. Biological Activity of Coriander Essential Oil

**Figure 2:**
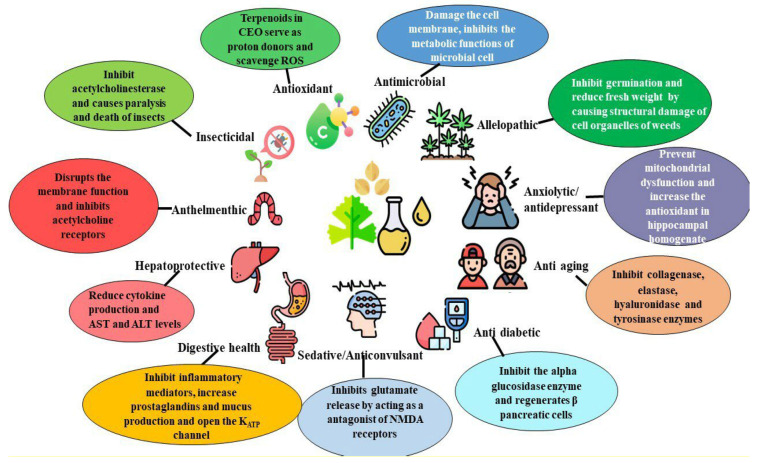
Biological properties of coriander essential oil.

At present, there has been a growing demand for the use of naturally-derived compounds in medicine, food preservation, pesticides, and herbicides. The coriander essential oils, produced as secondary metabolites, contain single or groups of phytocompounds that possess pharmacological activities such as antimicrobial, antioxidant, and insecticidal properties (Figure 2). The biological activities and therapeutic effects of coriander essential oil make it a suitable agent for treating bacterial and fungal infections in plants and animals, and for use in the pharmaceutical and food industries.

### 8.1. Antioxidant Activity

Oxidation of food such as meat, dairy and bakery products is one of the major causes of food spoilage, causing the loss of food quality and nutrition, which is a major loss for the food industry. Therefore, the food sector urgently needs new and efficient methods to help avoid food spoilage caused by oxidation [105]. Thus, coriander, as a spice, is used to season food in industries to prevent food spoilage. Coriander essential oil at a concentration of 0.1 g/mL is known to exhibit antioxidant properties by scavenging free radicals (DPPH and galvinoxyl) and inhibiting oxidative damage in lipid-containing foods [106]. It serves as a natural replacement for synthetic antioxidants such as BHA, BHT, TBHQ and propyl gallate used in the food industry. In the study conducted by Shahwar et al. [17], the antioxidant activity was evaluated using 1,1-Diphenyl-2-Picrylhydrazyl (DPPH) scavenging activity and Ferric-reducing antioxidant power assay (FRAP). In the DPPH assay, 500 μg of CSEO showed the highest radical-scavenging activity (RSA) of 66.48% when compared to 500 μg of CLEO which showed RSA of 56.73%. In the FRAP assay, the coriander seed oil showed an absorbance of 0.734, while coriander leaf oil showed an absorbance of 0.815. The terpenoid compounds present in coriander essential oil, such as linalool (monoterpene), are responsible for the antioxidant activity [107,108]. It bears hydroxyl groups that serve as proton donors that scavenge oxygen free radicals and act as inhibitors for radical chain reactions [98,109]. The antioxidant profile of *C. sativum* seed extract (mg/g dry weight) by Dua et al. [110] reports the presence of ascorbate, caffeic acid, ellagic acid, gallic acid, riboflavin, tocopherol, and polyphenol, as indicated in Table 6.

### 8.2. Antimicrobial Activity

Among the various naturally-occurring bioactive agents, plant essential oils have drawn attention as a potential source of antimicrobial compounds [6]. Coriander essential oil has shown antimicrobial activity against bacteria, fungi and virus by inhibiting their growth which will be discussed in detail in the upcoming sections. However, the activity varies with the compositions of the essential oils based on plant age, geographical regions, and oil extraction methods [115].

#### 8.2.1. Antibacterial Activity

The essential oils derived from various plant species have been previously investigated for antibacterial potential for application in food and pharmaceutical industries. The crude oil extract of *C. sativum* has shown antibacterial activity against Gram-negative and Gram-positive bacteria such as *Yersinia enterocolitica*, *Bacillus megaterium*, *Enterococcus faecalis*, *Escherichia coli*, *Klebsiella pneumoniae*, *Pseudomonas aeruginosa*, *Salmonella typhimurium*, *Listeria monocytogenes*, and *Staphylococcus aureus* [116,117]. EO derived from coriander has shown antibacterial activity against *Acinetobacter baumannii* LMG 1025 and LMG 1041 with MIC values of 1 µL/mL and 4 µL/mL respectively [118]. With minimum inhibitory concentration (MIC) values of 71.55, 86.4, and 35.2 g/mL, respectively, plantaricin, an antimicrobial peptide isolated from coriander leaf extract, exhibited antibacterial action against *K. pneumoniae*, *P. aeruginosa*, and *S. aureus* [119]. CEO permeabilizes the bacterial cell and damages the cell membrane, inhibits all the metabolic functions of the bacterial cell, and causes membrane polarization ultimately leading to bacterial cell death, and the linalool in the coriander essential oil exhibits its bactericidal action by acting on the bacterial cell wall [105,120]. The immunostimulant potential has elicited disease resistance in *Catla catla* fish infected with *Aeromonas hydrophila* [121]. The coriander EO has shown antibiofilm activity against *Stenotropomonas maltophilia* [122].

#### 8.2.2. Antifungal Activity

Plants are susceptible to fungal attack by direct contact or through wounds during growth. High moisture content on plant surfaces and high humidity are the major causes for fungal contamination. This could reduce the plant yield in agriculture and cause economic loss. However, some plants, such as coriander, produce essential oils that exhibit antifungal effects against pathogenic fungi [123]. Coriander EO has been used as a fungicide against fungi that are responsible for the spoilage of foods with a high moisture content. Thus, it is used in the food industry to inhibit fungal contamination in food. When the fungicidal activity of CEO was evaluated against *Candida* spp., it was observed that the minimum inhibition concentration (MIC) of CLEO ranged from 15.6–31.2 μg/mL, and the minimum fungicidal concentration (MFC) 31.2–62.5 μg/mL [124]. The growth of molds such as *Aspergillus niger*, *Penicillium expansum*, *Monilia sitophila*, *Penicillium stoloniferum* and *Rhizopus stolonifer* have been inhibited by the application of 0.15% CEO in cakes without affecting the quality of the product, as it was used in a minimal amount [98]. The application of coriander EO inhibited the growth of seed-borne pathogenic fungi such as *Alternaria alternata*, *Bipolaris oryzae*, *Curvularia lunata*, *Drechslera halodes*, *Fusarium oxysporum*, and *Tricoconis padwickii* in paddy [125]. In a study conducted by Soares et al., [126] coriander EO at 1 mg/mL concentration showed a zone of inhibition of 20–32 mm in *Microsporum canis* and 9–10 mm in *Candida* strains. CEO inhibits fungal growth by inhibiting the germ tube formation on yeast (*Candida* species). Furthermore, CEO permeabilizes the fungal membrane causing leakage of intracellular components [119].

### 8.3. Anthelmintic Activity

Gastroenteritis caused by parasitic worms in ruminants has been responsible for a decrease in animal productivity and farm profitability, and is recognized as one of the major challenges in livestock. Anthelmintic drugs such as albendazole and ivermectin are used to control and kill these parasitic helminths. However, the prevalence of multidrug-resistant parasites has hampered the effectiveness of these broad-spectrum anthelmintics. Thus, in order to reduce the accumulation of chemical residues and drug-resistant helminths, plant-derived extracts and oil serve as an alternative cost-effective approach. *C. sativum* extract has exhibited various medicinal properties and is also known to be effective against parasitic worms [127]. The anthelmintic activity of crude aqueous and hydro-alcoholic extracts of *C. sativum* fruit (0.5 mg/mL) has been observed to inhibit the hatching of eggs and act upon the adult nematode *Haemonchus contortus* [128]. In the study by Helal et al. [127], the anthelmintic effects of extracted coriander oil on third-stage larvae (L3s) of *Cooperia oncophora*, *Haemonchus contortus*, *Teladorsagia circumcincta*, *Trichostrongylus axei*, *Trichostrongylus colubriformis*, and *Trichostrongylus vitrinus* is examined. Coriander oil in combination with linalool had a synergistic anthelmintic effect by inducing structural damage in L3 larvae of all species except *C. oncophora*. With respect to plants, Sitophilus granarius, a parasite in chickpea grains, is inhibited by coriander EO [129]. The linalool in the coriander essential oil disrupts the membrane function through strong lipolytic activity and inhibits acetylcholine receptors, causing neurotoxicity in nematodes [127].

### 8.4. Insecticidal Activity

The storage of cereals and grains after harvest is prone to insect attack, incurring losses. Thus, proper storage of grains post-harvest is very crucial [130]. Synthetic insecticides such as carbon disulfide (CS_2_), methyl bromide (MB/MeBr/CH_3_Br), aluminum phosphide (AlP), carbon tetrachloride (CTC), acrylonitrile (ACN), ethylene dichloride (EDC), ethylene dibromide (EDB), and methyl benzoate are employed to protect and control the insect pest attack of stored goods, thus preventing post-harvest losses [131,132]. However, these synthetic insecticides are associated with safety concerns of the worker, insect resistance, accumulation of toxic residues in the food, environmental contamination, and the cost of treatment [133,134]. Currently, research has been focused on essential oils derived from plants for the protection of grains against pest attack, due to their insect repellent properties and fumigant activity [135]. The volatile toxicity of coriander EO against insects makes it a natural alternative to synthetic insecticides. The linalool fraction of coriander EO has been identified to be involved in controlling three rice pests: *Cryptolestes pusillus, Rhyzopertha dominica* and *Sitophilus oryzae* [136]. The essential oil has exhibited fumigant toxicity and repellent activity on pupae, larvae, and *Tribolium castaneum* [137], and the linalool has demonstrated high fumigant toxicity against *Lasioderma serricorne* [135]. The essential oils are potent neurotoxins that inhibit acetylcholinesterase in the central nervous system, causing hyperactivity, seizures, tremors and paralysis [138]. The mode of penetration of CEO into the insect cuticle and grain and its metabolic target is yet to be unveiled [137].

### 8.5. Antidiabetic Activity

Diabetes mellitus is a hyperglycemic condition caused by a decrease in insulin resistance, insulin secretion, or both. This metabolic disorder is responsible for chronic complications, which include neuropathic, macrovascular, and microvascular complications [139]. Despite the fact that the etiology of diabetes is still poorly understood, experimental studies reveal that reactive oxygen species could be one of the reasons for the pathogenesis of diabetes, which includes disruption of pancreatic beta cells and excess weight gain [140]. A number of medicines used to treat diabetes are available on the market, however, they come with certain drawbacks due to their high cost and side effects. Thus, medicinal plants are once again being researched for the treatment of diabetes, and Coriandrum sativum has been reported to exhibit antidiabetic activity [141]. In streptozotocin-induced diabetic mice, the antihyperglycaemic effect of coriander has been reported [142]. The effect of administration of coriander essential oil on streptozotocin-induced diabetic rats has been reported. In another study by Mahmoud et al. [143], the effect of coriander oil on dexamethasone-induced insulin resistance in rats has been studied. CEO could reverse the increase in glucose and insulin in serum, MDA and GSH levels in the pancreas, and BAX/BCL2 ratio induced by dexamethasone in rats. The linalool, geranyl acetate and γ-terpinene in coriander essential oil could significantly reduce serum glucose and increase glutathione peroxidase levels in diabetic mice [144]. CEO could inhibit the α-glucosidase enzyme which converts carbohydrates into monosaccharides in the small intestine before being absorbed. Furthermore, CEO lowers the blood glucose level by regenerating pancreatic beta cells (β cells) [139]. Linalool in CEO has restored the GLUT-1 expression in and has also enhanced the efficiency of rat diaphragm muscle in glucose uptake, improved glucose tolerance, and suppressed the formation of advanced glycation end products in diabetic rats [145,146].

### 8.6. Antihyperlipidemic/Hypolipidemic Activity

An increased level of fat product and its subsequent accumulation in subendothelial compartments of bone and vasculature results in hyperlipidemia. The excessive accumulation of fat could obstruct the blood flow, depriving tissues and organs such as the heart of oxygen-rich blood [147]. Many spices have been reported to exhibit hypolipidemic activity. Rich in petroselinic acid and other bioactive lipids, coriander seed oil has been thoroughly investigated for its hypolipidemic activity. In rats that had developed hyperlipidemia after being exposed to triton, coriander extract at a dosage of 1 g/kg decreased the absorption of lipids and increased the breakdown of lipids [148]. CEO decreased the triglycerides (TG), total cholesterol (TC) and HDL (High density lipoprotein) levels in serum that had been enhanced in rats treated with dexamethasone [143]. Coriander essential oil exhibited hypocholesterolemic properties in rats fed a cholesterol-rich diet by decreasing the levels of plasma total lipids (TL), total cholesterol (TC), triacylglycerols (TAG), and low-density lipoprotein-cholesterol (LDL-C) in plasma. Further, it has been shown that the high petroselinic acid (isomer of oleic acid) in coriander EO could suppress the level of arachidonic acid by mimicking the structure of the precursor of arachidonic acid and by inhibiting Δ6-desaturase [149]. Thus, coriander EO has scope to be used in the treatment of coronary heart diseases caused by high fat deposition.

### 8.7. Maintenance of Good Digestive Health

Nearly 10% of people worldwide suffer from peptic ulcers in the gastrointestinal tract, caused by a change in mucosal resistance. There are numerous synthetic drugs on the market to treat peptic ulcers, many of which have adverse side effects. Thus, peptic ulcers are currently treated with medicinal herbs rich in secondary metabolites [150]. Coriander is effective against *Helicobacter pylori* and thus helps in treating open sores in the inner mucosal lining of the mouth and stomach, preventing the formation of gastric ulcers. The antigastric activity in *C. sativum* is attributed to the antioxidants in the plant, which scavenge reactive oxygen species formed on the surface of gastric mucosa. Coriander extract also forms a protective hydrophilic layer against gastric injury [21]. The phytocompounds such as carvacrol, terpineol, elemol present in the essential oil inhibit inflammatory mediators, increase prostaglandins, increase mucus production and open the KATP channel, thus healing gastric lesions [151]. These compounds are present in coriander essential oil and can thus be used in treating peptic ulcers. In a study by Heidari et al. [152], the protective effects of coriander essential oil against colitis induced by 4% acetic acid in Wistar rats are examined. Doses of 0.5 and 1 mL of the essential oil reduced the colon’s weight and were effective against lesions caused by colitis. The mode of action of CEO in treating colitis is by inhibiting cytokines and the pro-inflammatory mediator expression by suppressing necrosis factor (NF)- κ B activation in macrophages. The herb oil is actively involved in the elimination of toxins internally, and maintains good digestive health. This can be achieved by the topical application of coriander EO along with a carrier oil on the belly and lower back area [21].

### 8.8. Hepatoprotective Activity

The liver, a primary site of detoxification and metabolism of xenobiotics, is prone to frequent injury by drugs, toxic chemicals, and infiltrated microbes [153]. Treatments with naturally-derived drugs for hepatotoxicity are preferable over synthetic drugs, which would otherwise cause adverse side effects. Hepatotoxicity is associated with the generation of reactive oxygen species in the liver. Plants rich in antioxidant activity offer protection against reactive oxygen species. Essential oils from medicinal plants have been a suitable replacement for surgery, pharmacotherapy, and liver transplantation, all of which come with major complications [154]. Essential oils could reduce the cytokine production in hepatic injury-associated inflammation [155].

Coriander essential oil has been checked for its hepatoprotective activity against carbon tetrachloride (CCl_4_) in rats. The levels of aspartate aminotransferase (AST) and alanine aminotransferase (ALT) in the liver were analyzed, and the histopathology of carbon tetrachloride-treated mice was also performed. The essential oil reduced AST levels and ALT levels in the blood and exhibited hepatoprotective activity [156]. The reduction in AST and ALT level is attributed to α-pinene, camphor, geraniol, geranyl acetate, γ-terpinene, linalool, and *p*-cymene present in the essential oil. However, the linalool present in CEO is considered as a potential therapeutic agent against CCl_4_-induced hepatic damage [157,158,159]. The linalool inhibits the expression and production of inflammatory mediators induced by CCl_4_ and ameliorates against CCl_4_ induced hepatotoxicity [158]

### 8.9. Anti-Aging Properties

An essential part of the epidermis of skin is long chain fatty acid that maintains the structure and function of the human skin. However, over time, the loss of fatty acids in the epidermis results in aging, which has become an individual’s primary concern [160]. Considering the expensive cost of high-quality anti-aging products, it is essential to look for other, more affordable alternatives. Once again, plant-derived products are the most suitable replacement for costly anti-aging products. Coriander oil can be applied topically to promote skin healing and is a great substitute for sunscreen. In the study by Salem et al. [161] coriander essential oil was evaluated for antiwrinkle cosmetic potential activity in UV-induced skin photoaging mice. It was observed that coriander essential oil showed the highest collagenase, elastase, hyaluronidase and tyrosinase inhibitory activities. This is attributed to the inhibition of numerous cell-signaling pathways, downregulation of mRNA expression of MMP-1, decrease in expression of AP-1, COX-2, JNK, MDA, and PGE-2 levels, and increase in expression of SMAD3, TGFβ, and TGFβII levels. Furthermore, the linalool present in coriander oil exhibited antioxidative effects, preventing ROS generation caused by UV exposure. However, very few studies have reported the anti-aging effects of coriander essential oil on skin, and thus, there is scope for further clinical studies into its extensive application in the cosmetic industry.

### 8.10. Sedative/Anticonvulsant Properties

Nearly 20–30% of the human population suffers from epilepsy, a neurological disorder associated with seizures caused by abnormal brain activity. Currently, there are very few antiepileptic drugs (AEDs) available that can control seizures. However, epilepsy comes together with psychological disorders such as anxiety and depression. Opting for surgery as an option to control epilepsy only targets single localizable sites where seizures originate. Thus, there is a need for novel drug therapies that can efficiently act against drug-resistant seizures, which pose no side effects (psychiatric effects), are cheap, and are easily available. Currently, a number of studies identify natural medicines as potential antiepileptic drugs, and essential oils having distinctive chemical characteristics that make them suitable candidates for designing antiepileptic drugs [162]. Coriander EO, a rich source of linalool, has shown sedative and anticonvulsant properties.

A study conducted by Gastón et al. [163] assessed the effects of intracerebroventricular (ICV) administration of CEO at 0.86, 8.6 and 86 μg/chic doses on emotionality and locomotor activity in neonatal chicks. The ICV injection of coriander induced a sedative effect at 8.6 and 86 μg doses, where the number of defecations, vocalizations, and escape attempts decreased and sleeping posture increased significantly. Earlier the study conducted by Emamghoreishi and Heidari-Hamedani [164] also showed the anticonvulsant properties of aqueous and hydroalcoholic extracts of CEO (200, 400, 600, and 800 mg/kg dose) against convulsion induced by pentylenetetrazole (PTZ). The highest activity (86% reduction in mortality) was achieved at a dosage of 800 mg/kg. The linalool present in CEO acts as a sedative by inhibiting glutamate release by acting as a competitive antagonist of ionotropic receptors of N-methyl-d-aspartate (NMDA) that mediate excitatory neurotransmission in the CNS [163,165].

### 8.11. Anxiolytic-Antidepressant Properties

Anxiety disorders, such as generalized and social anxiety disorder, obsessive–compulsive disorder (OCD), and post-traumatic stress disorder (PTSD), are one of the most frequent psychological issues around the globe, affecting nearly 40 million people in the U.S. [166]. Anxiety disorders are currently treated with psychotherapy or antidepressant medications, such as benzodiazepine anti-anxiety drugs, monoamine oxidase inhibitors, and serotonin tricyclics, which have life-threatening side effects. Various essential oils like *Citrus limon*, *Citrus sinensis*, *Rosa damascena*, and *Santalum album* are known to relieve anxiety and stress [167]. The anxiolytic and antidepressant properties of coriander essential oil have been studied in vivo in β-amyloid rat models with Alzheimer’s disease by Cioanca et al. [168]. CEO enhanced the locomotor activity, increased the time spent and the number of entries in the open arm within the elevated plus-maze test, and also increased the time for swimming and immobility within the forced swimming test. Thus, CEO proved to be an excellent medicine for treating the pathophysiology of Alzheimer’s disease.

The terpenes could prevent mitochondrial dysfunction and oxidative stress [40]. Furthermore, both doses of coriander volatile oil (CO1% and CO3%), restored the activity of CAT (Catalase) and increased GSH (glutathione) levels in the hippocampal homogenates of Aβ (1–42)-treated rats. These results suggest an increase in anxiolytic and antidepressant-like behaviors, along with an increase in the antioxidant status in the hippocampal homogenates. The antidepressant activities of coriander oil are mediated by the action of the GABA receptor complex [168].

### 8.12. Allelopathy

One of the major challenges in agriculture is pest attacks and overgrowing weeds that compete with the main crop plants for nutrients and water uptake, resulting in poor crop yield [169,170]. This is controlled by the application of synthetic herbicides and pesticides in order to increase the crop yield [171]. However, continuous use of these herbicides and pesticides can result in herbicide-resistant weeds and environmental contamination. Thus, synthetic herbicides and pesticides can be replaced with natural plant-derived compounds for a safe environment and to produce high quality crops. Most aromatic plants release chemical compounds called allelochemicals that are phytotoxic to receiving organisms (weeds), and can be applied to kill plant pests. The essential oil in coriander is allelopathic and can be exploited as a biological agent to resist plant pests and reduce the growth of weeds [172,173].

Coriander essential oil is reported to show allelopathic activity against a wide range of weeds. In a study conducted by Azirak and Karaman, (2008), 3, 6, 10, and 20 µL has been used against various weed species such as *Amaranthus retroflexus* L., *Raphanus raphanistrum* L., *Alcea pallida* Waldst. & Kit., *Avena fatua*, *Sinapis arvensis* L., *Centaurea salsotitialis* L., *Rumex nepalensis* Spreng., and *Sonchus oleraceus* L. Doses of 10 and 20 µL of EO successfully inhibited the germination of all of these weed species except for *Raphanus* seeds [174]. When grown along with coriander, the EO released from coriander reduced the fresh weight of barnyardgrass, black nightshade, common lambsquarters, and purslane [175]. The germination of two weeds, *Lathyrus annuus* and *Vicia villosa*, was completely inhibited by coriander seed EO at 200–800 ppm [176]. The mode of action of these essential oils against weeds is still unclear. However, it is presumed that the monoterpenes present in the essential oils would have caused internal structural damage to cells causing the breakdown and decomposition of intact organelles within the cell [174].

## 9. Conclusions and Prospects

Herbal medicines derived from medicinal and aromatic plants have been used as a safe therapeutic modality for thousands of years. The essential oils derived from plants exhibit various pharmacological properties, which can be attributed to the bioactive compounds present in the essential oils. The importance of the use of coriander essential oil derived from *Coriandrum sativum* as an alternative to synthetic drugs has been discussed in this study. The phytochemical composition of the oil, and the percentage of each of the phytochemical compounds in the coriander leaf and seed oil has been listed. A comparison on the variation of the various phytochemicals in the essential oils across different germplasms and different geographical locations has been made in the current review. The phytochemical composition helps gain a better understanding of the mode of action of CEO against various ailments. The pharmacological properties of CEO, such as antimicrobial properties against bacteria and fungi, antioxidant activity, antidiabetic, anxiolytic activity, anthelmintic, insecticidal activity, sedative, digestive, hepatoprotective and anti-aging properties have been discussed. This essential oil is not just limited to medicine but also finds its application in the food and cosmetic industries. Furthermore, due to its allelopathic properties, it has been utilized in pest and weed management in agriculture. It is the most promising replacement for chemical-based herbicides and pesticides, causing less environmental damage and wide public acceptance. With these applications, there is a need for large-scale production of essential oils.

The production and extraction of essential oils would demand extensive harvesting of plants. Thus, there is scope for the development of elite germplasms with high oil-yielding capacity and resistance to biotic and abiotic stress by using sequencing technologies, multi-omics approach (transcriptomics, proteomics, metabolomics, and ionomics), Genome-Wide Association Studies (GWAS), and genomic selection. Linalool, one of the major phytochemicals present in the essential oil confers various pharmacological properties to CEO. Thus, the linalool content in the plant can be enhanced by the overexpression of genes involved in linalool synthesis (monoterpene biosynthesis pathway), and linalool accumulation (*CsLINS*), through metabolic engineering. The synergistic effect of coriander essential oil in combination with other essential oils, or of CEO in combination with its individual phytocompounds (such as Linalool), has to be explored, and the application of this combination in the food industry, cosmetic industry and international pest management (IPM) industry has to be considered. Further, there is a need for extensive validation of the true efficacy and safety of these plant-derived products such as CEO and its individual phytocompounds (linalool) through well-designed clinical trials. The efficacy and the biological properties of coriander essential oils, which are prone to undergo oxidation, can be retained through nanotechnological approaches. Studies must be conducted on the nanoencapsulation of CEO in drug delivery systems in order to enhance the solubility, stability and efficacy of essential oil-based formulations.

## Figures and Tables

**Table 1:** The chemical composition of essential oil of coriander [27].

Chemical Group	Constituents
Alcohols	Linalool (60–80%), geraniol (1.2–4.6%), terpinen-4-ol (3%), α-terpineol (0.5%)
Hydrocarbons	Limonene (0.5–4.0%), g-terpinene (1–8%), *p*-cymene (3.5%), α-pinene (0.2–8.5%), camphene (1.4%), myrcene (0.2–2.0%)
Ketones	Camphor (0.9–4.9%)
Esters	Geranyl acetate (0.1–4.7%), linalyl acetate (0–2.7%)

**Table 2:** Chemical composition of coriander seed and leaf oil along with its biological significance.

Constituent	Concentration (%)		Function	References
	Seed	Leaf		
Linalool	40.9−79.9	0–13	Antimicrobial, anti-inflammatory, anticancer, antioxidant properties	[30,31]
Geraniol	0.5–3.0	-	Insecticidal and repellent properties	[32]
Terpinen-4-ol	0.43–1	0.2	Promotes anti-inflammatory cytokine production, enhances the effect of several chemotherapeutic and biological agents	[33]
α-terpineol	0.5–1.5	-	Anticancer, anticonvulsant, antiulcer, antihypertensive	[34]
γ-terpinene	0.3–11.2	0.9–3.1	Potential biofuel alternative	[35]
*p*-cymene	0.5–1.5	-	Natural antioxidant, antimicrobial activity	[36]
Limonene	2.0–5.0	-	Anticancer activity	[37]
α-pinene	0.2–10.9	1.9–2.5	Antimicrobial, apoptotic, antimetastatic, and antibiotic	[38]
Camphene	1.3–2	-	Antileishmanial, hepatoprotective, antiviral and anticancer activity by inducing apoptosis in cancer cells	[39]
Myrcene	0.5–1.5	-	Anxiolytic, antioxidant, anti-ageing, anti-inflammatory, analgesic properties	[40]
Camphor	0.9–4.9	-	Insecticidal, antimicrobial, antiviral, anticoccidial, antinociceptive	[41]
Geranyl acetate	0.2–5.4	-	Antinociceptive activity	[17,42]
Linalyl acetate	0–2.7	-	Flavoring agent, antimicrobial and anti-inflammatory activity	[43]
Eucalyptol	0.1–1%	0.5–2	Anti-inflammatory and antioxidant mainly via the regulation on NF-κB and Nrf2 pathway	[44]
β-phellandrene	0–1.5	-	Antimicrobial activity	[45]
Borneol	4.5	-	Acesodyne, sedation, anti-inflammation, antibiosis effect	[46]
β-caryophyllene	3.26	-	Antibacterial, antioxidant, gastroprotective, anxiolytic, anti-inflammatory	[47]
Citronellol	0.15–0.25	8.1–10	Anti-inflammatory, analgesic and anticonvulsant effects	[48]
Caryophyllene oxide	3.12	-	Cytotoxic activity, analgesic activity	[49]
Thymol	2.4–3	-	Antioxidant and antimicrobial properties	[50]
Decanal	-	5.1–8.8	Antioxidant and antimicrobial properties	[17]
(E)-2-Dodecenal	-	12–14	Anthelmintic activity	[51]
Tridecanal	-	0.3–0.4	Antibacterial, antifungal and antioxidant activities	[52]
1-Decanol	-	5–10%	Bactericidal activity and membrane-damaging activity	[53]
(E)-2-Tetradecenal	-	4.1–8.2	Antimicrobial, and antioxidant activity	[54]
(E)-2-Decenal	-	20–35	Antimicrobial, and antioxidant activities	[55]
2-decenoic acid	-	30.8	Antimicrobial activity	[56]
Capric acid	-	12.7	Antibacterial and anti-inflammatory activity	[57]

**Table 3:** Variation of coriander essential oil among different varieties of coriander.

Location	Varieties	Essential Oil Content (%)	Linalool Content (%)	References
Turkey	Arslan	0.30	89.46	[60]
	Gürbüz	0.33	89.44	
	Erbaa	0.38	91.77	
	Gamze	0.35	89.77	
Pakistan	Native	0.15	69.64	[20]
Egypt	Russian	0.19	69.50	[61]
	Balady	0.2	62.53	
	Selected	0.09	65.48	
India	Acr1	0.25	78.22	[62]
	Sindhu	0.32	81.3	
	Swathi	0.34	73.2	
	Sadhna	0.29	79.2	
	Sudha	0.32	85.3	

**Table 4:** Variation of some important phytochemicals in coriander seed EO across different geographical locations.

	Percentage of Important Phytochemicals in Coriander Seed Oil from Different Geographical Locations									
Constituent	Bangladesh [28]	Bulgaria [77]	India [78]	Iran [58,71]	Morocco [79]	Pakistan [20]	Poland [80]	Romania [81]	Russia [82]	Turkey [60,83]
α-Thujene	-	-	-	-	0.10	0.02	-	-	-	-
α-Pinene	-	7.14	2.81	3.3	7.69	1.63	5.03	1.62	6.44	3.06
α-Terpineol	-	0.66	-	-	0.54	-	0.63	-	0.81	-
β-Pinene	1.8	1.15	0.48	0.4	0.93	0.23	0.48	0.71	0.34	0.25
β-caryophyllene	0.3	0.17	-	-	0.14	0.07	-	0.44	-	-
*p*-Cymene	1.3	2.67	0.42	2	0.03	1.12	0.54	8.000	7.44	0.39
γ-Terpinene	14.4	7.35	0.15	9.3	11.59	4.17	3.80	5.236	-	0.20
Borneol	0.3	0.44	0.14	-	0.42	0.18	-	-	-	-
Camphor	-	8.30	-	0.2	6.98	0.38	3.90	6.01	7.94	3.56
Camphene	-	2.37	-		1.07		0.64		1.09	-
Citronella	1.3	-	-	0.3	-	0.65	-	-	-	-
Decanal	0.1	-	0.20	0.4	0.13	0.14	-	-	-	0.54
Geraniol	1.9	-	24.51	1.9	2.79	-	1.07		0.11	2.37
Geranyl acetate	17.6	5.05		4.0	-	4.99	2.13	1.423	3.19	2.77
Limonene	0.4	5.19		0.3	3.24	0.26	2.58	9.628	3.29	0.27
Linalool	37.7	50.16	57.52	70.1	48.41	69.60	78.45	45.387	59.92	75.26
Myrcene	0.6	1.78	0.37	0.2	1.16	0.18	0.47	1.504	0.20	0.42
Neryl acetate	-	-		6.9	6.47	-	-	-	-	-
Sabinene	0.2	0.73		0.2	0.58	0.12	-	-	-	0.10
Thymol	-	-		0.2	0.06	0.41	-	0.376	-	-
Undecanal	0.1	-	0.13	-	0.06	0.41	-	-	-	-

**Table 5:** Extraction and analysis of EO in coriander.

Extraction Method	Extraction Condition	Quantification Method	Quantification Method (Instrument Used, Model, Conditions)	Quantity of Oil Obtained in %	References
Hydrodistillation	80 min	GC-MS	Turbomass system,199 °C for 35 min, 1 μL	0.18–1.4	[59]
	180 min	GC-MS	ITMS Varian 4000 GC-MS/MS 250 °C for 10 min, 1 μL	0.29	[93]
	180 min	GC-MS	Shimadzu GC-9A, Varian 3400, 250 °C	0.31	[22]
Microwave-assisted hydrodistillation	60 min with 500 W power	-	-	0.32	[94]
	240 min	GC-MS, FID	Shimadzu 15A 260 °C, 1 mL/min	0.1	[89]
Rapid solid–liquid dynamic extraction	8 bar, 6 h	solid-phase microextraction/gas chromatography coupled to mass spectrometry	Clarus 580 GC apparatus coupled to a Clarus SQ 8 S GC/MS, 250 °C	0.73	[95]
Soxhlet extraction	40 °C	GC-MS and GC-FID	Agilent GC890N 150 °C, 5 μL	14.45	[91]
	Methylene chloride	GC-MS	Agilent Technologies series 6890N/5975B 280 °C, 20 min, 1 μL	5.10	[96]
	Petroleum ether, 45 min	GC-MS	Shimadzu, GCMS-QP2010 Ultra, 260 °C	8.82	[97]
Steam distillation	80 min	GC-MS, FID	Agilent Technologies 6890, 340 °C,		[98]
Subcritical water extraction	125 °C, 0.5 mm, and 2 mL/min	GC-FID and GC-MS	Phillips model PU-4500 50 to 240 C at 3 C/min, 0.5 μL	14.1	[99]
	10 °C min^−1^ to 200 °C, 15 min	GC-MS	Agilent gas chromatography model 6890N 200 °C, 15 min	0.6–0.8	[100]
extraction	10 MPa, 35 °C, CO_2_ - 419.9 kg/m^3^	GC-FID and GC-MS	capillary type HP 5890 series II, equipped with a DB-5MS column 280 °C for 53 min, 0.5 μL	0.84	[101]
	300 bar, 35 °C,	GC	Shimadzu Model RF-353, 50–200 °C, 5 μL	20	[102]
	50 °C and 150 bar, 180 min	GC-MS	Fisons Instruments MD 800, 250 °C, 20 min, 0.4 μL	0.61	[103]
	350 bar, 35 °C, CO_2_ -14 g/min	GC-MS	Shimadzu QP2010 Ultra, 280 °C, 30 min	4.55	[104]

Note: GC-MS: Gas chromatography-mass spectroscopy; FID: Flame ionization detector.

**Table 6:** Antioxidant profile of *C. sativum* seed extract.

Metabolite	Amount in mg/g Dry Weight of Sample	Method of Analysis	References
Caffeic Acid	0.08	HPLC method	[111]
Ellagic Acid	0.162	HPLC method	[111]
Gallic Acid	0.173	HPLC method	[111]
Kaempferol	0.233	HPLC method	[111]
Oxidized Ascorbate	0.15	Spectrophotometric method	[112]
Reduced Ascorbate	0.136	Spectrophotometric method	[112]
Riboflavin	0.0046	Spectrophotometric method	[113]
Tocopherol	0.181	Spectrophotometric method	[113]
Total Ascorbate	0.287	Spectrophotometric method	[112]
Total Polyphenol	18.7	Folin-Ciocalteau method	[114]
Quercetin	0.608	HPLC method	[111]

## Data Availability

Not applicable.

## References

[1] Wu Y., Luo Y., Wang Q. (2012). Antioxidant and antimicrobial properties of essential oils encapsulated in Zein nanoparticles prepared by liquid–liquid dispersion method. LWT-Food Sci. Technol..

[2] Mandal S., Mandal M. (2015). Coriander (*Coriandrum sativum* L.) essential oil: Chemistry and biological activity. Asian Pac. J. Trop. Biomed..

[3] Guenther E. (1948). The essential oils. J. Am. Pharm. Assoc..

[4] CheBi Volatile Oil Component (CHEBI:27311). https://www.ebi.ac.uk/chebi/searchId.do?chebiId=CHEBI:27311.

[5] Rowan D.D. (2011). Volatile metabolites. Metabolites.

[6] Hyldgaard M., Mygind T., Meyer R.L. (2012). Essential oils in food preservation: Mode of action, synergies, and interactions with food matrix components. Front. Microbiol..

[7] Yee L.L., Phebe D. (2016). Physiological production of essential oil in plants-Ontogeny, secretory structures and seasonal variations: Review. Pertanika J. Sci. Technol..

[8] Sharma P.R., Mondhe D.M., Muthiah S., Pal H.C., Shahi A.K., Saxena A.K., Qazi G.N. (2009). Anticancer activity of an essential oil from *Cymbopogon flexuosus*. Chem. Biol. Interact..

[9] Passos G.F., Fernandes E.S., da Cunha F.M., Ferreira J., Pianowski L.F., Campos M.M., Calixto J.B. (2007). Anti-inflammatory and anti-allergic properties of the essential oil and active compounds from *Cordia verbenacea*. J. Ethnopharmacol..

[10] Gkogka E., Hazeleger W.C., Posthumus M.A., Beumer R.R. (2013). The antimicrobial activity of the essential oil of *Pistacia lentiscus* var. Chia. J. Essent. Oil-Bear. Plants..

[11] Freires I.A., Denny C., Benso B., de Alencar S.M., Rosalen P.L. (2015). Antibacterial activity of essential oils and their isolated constituents against cariogenic bacteria: A systematic review. Molecules.

[12] Diederichsen A., Hammer K. (2003). The infraspecific taxa of coriander (*Coriandrum sativum* L.). Genet. Resour. Crop Evol..

[13] Khan I.U., Dubey W., Gupta V. (2014). Taxonomical aspect of coriander (*Coriandrum sativum* L.). Int. J. Curr. Res. Rev..

[14] Yeung E.C., Bowra S. (2011). Embryo and endosperm development in coriander (*Coriandrum sativum*). Botany.

[15] Gardenate Growing Coriander. https://www.gardenate.com/plant/Coriander?page=1&co=ALL.

[16] Ware M. Cilantro (Coriander): Benefits, nutrition, and preparation tips. https://www.medicalnewstoday.com/articles/277627.

[17] Shahwar M.K., El-Ghorab A.H., Anjum F.M., Butt M.S., Hussain S., Nadeem M. (2012). Characterization of coriander (*Coriandrum sativum* L.) seeds and leaves: Volatile and non-volatile extracts. Int. J. Food Prop..

[18] Chahal K.K., Singh R., Kumar A., Bhardwaj U. (2016). Chemical composition and biological activity of *Coriandrum sativum* L.: A review. IOSR J. Pharm..

[19] Sarkic A., Stappen I. (2018). Essential Oils and their single compounds in cosmetics—A critical review. Cosmetics.

[20] Farooq A., Muhammad S., Abdullah I.H., Nazamid S., Shahid I., Umer R. (2011). Physicochemical composition of hydro-distilled essential oil from coriander (*Coriandrum sativum* L.) seeds cultivated in Pakistan. J. Med. Arom. Plant Sci..

[21] SurgingLife Coriander Essential Oil Its Uses and What It Is. https://surginglife.com/wellness/essential-oils/guide/coriander/.

[22] Wahba H.E., Abd Rabbu H.S., Ibrahim M.E. (2020). Evaluation of essential oil isolated from dry coriander seeds and recycling of the plant waste under different storage conditions. Bull. Natl. Res. Cent..

[23] Wei J.-N., Liu Z.-H., Zhao Y.-P., Zhao L.-L., Xue T.-K., Lan Q.-K. (2019). Phytochemical and bioactive profile of *Coriandrum sativum* L.. Food Chem..

[24] Morsy N.F.S. (2017). Chemical structure, quality indices and bioactivity of essential oil constituents. Active Ingredients from Aromatic And Medicinal Plants.

[25] Burdock G.A., Carabin I.G. (2009). Safety assessment of coriander (*Coriandrum sativum* L.) essential oil as a food ingredient. Food Chem. Toxicol..

[26] An Q., Ren J.-N., Li X., Fan G., Qu S.-S., Song Y., Li Y., Pan S.-Y. (2021). Recent updates on bioactive properties of linalool. Food Funct..

[27] Nadeem M., Faqir M.A., Muhammad I.K., Tehseen S., El-Ghorab A., Javed I.S. (2013). Nutritional and medicinal aspects of coriander (*Coriandrum sativum* L.): A Review. Br. Food J..

[28] Bhuiyan M.N.I., Begum J., Sultana M. (2009). Chemical composition of leaf and seed essential oil of *Coriandrum sativum* L. from Bangladesh. Bangladesh J. Pharmacol..

[29] Chung I.-M., Ahmad A., Kim S.-J., Naik P.M., Nagella P. (2012). Composition of the essential oil constituents from leaves and stems of korean *Coriandrum sativum* and their immunotoxicity activity on the *Aedes aegypti* L.. Immunopharmacol. Immunotoxicol..

[30] Kamatou G.P.P., Viljoen A.M. (2008). Linalool–A review of a biologically active compound of commercial importance. Nat. Prod. Commun..

[31] Satyal P., Setzer W.N. (2020). Chemical compositions of commercial essential oils from *Coriandrum sativum* fruits and aerial parts. Nat. Prod. Commun..

[32] Chen W., Viljoen A.M. (2010). Geraniol—A review of a commercially important fragrance material. S. Afr. J. Bot..

[33] Shapira S., Pleban S., Kazanov D., Tirosh P., Arber N. (2016). Terpinen-4-Ol: A novel and promising therapeutic agent for human gastrointestinal cancers. PLoS ONE.

[34] Khaleel C., Tabanca N., Buchbauer G. (2018). α-Terpineol, a natural monoterpene: A review of its biological properties. Open Chem..

[35] Qi C., Zhao H., Li W., Li X., Xiang H., Zhang G., Liu H., Wang Q., Wang Y., Xian M. (2018). Production of γ-terpinene by metabolically engineered *Escherichia coli* using glycerol as feedstock. RSC Adv..

[36] Marchese A., Arciola C.R., Barbieri R., Silva A.S., Nabavi S.F., Tsetegho Sokeng A.J., Izadi M., Jafari N.J., Suntar I., Daglia M. (2017). Update on monoterpenes as antimicrobial agents: A particular focus on *p*-cymene. Materials.

[37] Mukhtar Y.M., Adu-Frimpong M., Xu X., Yu J. (2018). Biochemical significance of limonene and its metabolites: Future prospects for designing and developing highly potent anticancer drugs. Biosci. Rep..

[38] Rivas da Silva A.C., Lopes P.M., Barros de Azevedo M.M., Costa D.C.M., Alviano C.S., Alviano D.S. (2012). biological activities of α-pinene and β-pinene enantiomers. Molecules.

[39] Vallianou I., Hadzopoulou-Cladaras M. (2016). Camphene, a plant derived monoterpene, exerts its hypolipidemic action by affecting srebp-1 and mtp expression. PLoS ONE.

[40] Surendran S., Qassadi F., Surendran G., Lilley D., Heinrich M. (2021). Myrcene-what are the potential health benefits of this flavouring and aroma agent?. Front. Nutr..

[41] Chen W., Vermaak I., Viljoen A. (2013). Camphor-a fumigant during the black death and a coveted fragrant wood in ancient Egypt and Babylon-a review. Molecules.

[42] Quintans-Júnior L., Moreira J.C.F., Pasquali M.A.B., Rabie S.M.S., Pires A.S., Schröder R., Rabelo T.K., Santos J.P.A., Lima P.S.S., Cavalcanti S.C.H. (2013). Antinociceptive activity and redox profile of the monoterpenes (+)-camphene, *p*-cymene, and geranyl acetate in experimental models. ISRN Toxicol.

[43] Peana A.T., D’Aquila P.S., Panin F., Serra G., Pippia P., Moretti M.D.L. (2002). Anti-inflammatory activity of linalool and linalyl acetate constituents of essential oils. Phytomedicine.

[44] Cai Z.-M., Peng J.-Q., Chen Y., Tao L., Zhang Y.-Y., Fu L.-Y., Long Q.-D., Shen X.-C. (2021). 1,8-Cineole: A review of source, biological activities, and application. J. Asian Nat. Prod. Res..

[45] Zhang J.-H., Sun H.-L., Chen S.-Y., Zeng L., Wang T.-T. (2017). Anti-fungal activity, mechanism studies on α-phellandrene and nonanal against *Penicillium cyclopium*. Bot. Stud..

[46] Chen N., Wen J., Wang Z., Wang J. (2022). Multiple regulation and targeting effects of borneol in the neurovascular unit in neurodegenerative diseases. Basic Clin. Pharmacol. Toxicol..

[47] Francomano F., Caruso A., Barbarossa A., Fazio A., La Torre C., Ceramella J., Mallamaci R., Saturnino C., Iacopetta D., Sinicropi M.S. (2019). β-caryophyllene: A sesquiterpene with countless biological properties. NATO Adv. Sci. Inst. Ser. E Appl. Sci..

[48] Santos P.L., Matos J.P.S.C.F., Picot L., Almeida J.R.G.S., Quintans J.S.S., Quintans-Júnior L.J. (2019). citronellol, a monoterpene alcohol with promising pharmacological activities-a systematic review. Food Chem. Toxicol..

[49] Fidyt K., Fiedorowicz A., Strządała L., Szumny A. (2016). β-caryophyllene and β-caryophyllene oxide-natural compounds of anticancer and analgesic properties. Cancer Med..

[50] Escobar A., Pérez M., Romanelli G., Blustein G. (2020). Thymol bioactivity: A review focusing on practical applications. Arab. J. Chem..

[51] Forbes W.M., Gallimore W.A., Mansingh A., Reese P.B., Robinson R.D. (2014). Eryngial (trans-2-Dodecenal), a bioactive compound from eryngium foetidum: Its identification, chemical isolation, characterization and comparison with Ivermectin in vitro. Parasitology.

[52] Silva C.A.M., Simeoni L.A., Silveira D. (2009). Genus *Pouteria*: Chemistry and biological activity. Rev. Bras. Farmacogn..

[53] Togashi N., Shiraishi A., Nishizaka M., Matsuoka K., Endo K., Hamashima H., Inoue Y. (2007). Antibacterial activity of long-chain fatty alcohols against *Staphylococcus aureus*. Molecules.

[54] Casiglia S., Bruno M., Rosselli S., Senatore F. (2016). Chemical composition and antimicrobial activity of the essential oil from flowers of *Eryngium triquetrum* (apiaceae) collected wild in Sicily. Nat. Prod. Commun..

[55] Trombetta D., Saija A., Bisignano G., Arena S., Caruso S., Mazzanti G., Uccella N., Castelli F. (2002). Study on the mechanisms of the antibacterial action of some plant alpha, beta-unsaturated aldehydes. Lett. Appl. Microbiol..

[56] Marques C.N.H., Morozov A., Planzos P., Zelaya H.M. (2014). The fatty acid signaling molecule cis-2-decenoic acid increases metabolic activity and reverts persister cells to an antimicrobial-susceptible state. Appl. Environ. Microbiol..

[57] Huang W.-C., Tsai T.-H., Chuang L.-T., Li Y.-Y., Zouboulis C.C., Tsai P.-J. (2014). Anti-bacterial and anti-inflammatory properties of capric acid against *Propionibacterium* acnes: A comparative study with lauric acid. J. Dermatol. Sci..

[58] Nejad E.S., Hadian J., Ranjbar H. (2010). Essential oil compositions of different accessions of *Coriandrum sativum* L. from Iran. Nat. Prod. Res..

[59] Zheljazkov V.D., Pickett K.M., Caldwell C.D., Pincock J.A., Roberts J.C., Mapplebeck L. (2008). Cultivar and sowing date effects on seed yield and oil composition of coriander in Atlantic Canada. Ind. Crops Prod..

[60] Beyzi E., Karaman K., Gunes A., BuyukkilicBeyzi S. (2017). Change in some biochemical and bioactive properties and essential oil composition of coriander seed (*Coriandrum sativum* L.) varieties from Turkey. Ind. Crops Prod..

[61] Abou El-Nasr T.H.S., Ibrahim M.M., Aboud K.A., El-Enany M.A. (2013). Assessment of genetic variability for three coriander (*Coriandrum sativum* L.) cultivars grown in Egypt, using morphological characters, essential oil composition and ISSR markers. World Appl. Sci. J..

[62] Saxena S.N., Rathore S.S., Saxena R., Barnwal P., Sharma L.K., Singh B. (2014). Effect of cryogenic grinding on essential oil constituents of coriander (*Coriandrum sativum* l.) genotypes. J. Essent. Oil-Bear. Plants.

[63] López P.A., Widrlechner M.P., Simon P.W., Rai S., Boylston T.D., Isbell T.A., Bailey T.B., Gardner C.A., Wilson L.A. (2008). Assessing phenotypic, biochemical, and molecular diversity in coriander (*Coriandrum sativum* L.) germplasm. Genet. Resour. Crop Evol..

[64] Saxena S.N., Swarup Meena R., Vishal M.K., John S., Kumar Sharma L., Mishra B.K., Agarwal D. (2022). Variation in essential oil constituents of coriander (*Coriandrum sativum* L.) germplasm across coriander growing regions in India. J. Essent. Oil Res..

[65] Sampaio T.S., Nogueira P.C.L. (2006). Volatile components of Mangaba fruit (*Hancornia speciosa* Gomes) at three stages of maturity. Food Chem..

[66] Visai C., Vanoli M. (1997). Volatile compound production during growth and ripening of peaches and nectarines. Sci. Hortic..

[67] Vendramini A.L., Trugo L.C. (2000). Chemical composition of acerola fruit (*Malpighia punicifolia* L.) at three stages of maturity. Food Chem..

[68] Nguyen Q.-H., Talou T., Evon P., Cerny M., Merah O. (2020). Fatty acid composition and oil content during coriander fruit development. Food Chem..

[69] Msaada K., Hosni K., Taarit M.B., Chahed T., Kchouk M.E., Marzouk B. (2007). Changes on essential oil composition of coriander (*Coriandrum sativum* l.) fruits during three stages of maturity. Food Chem..

[70] Telci I., Bayram E., Avci B. (2006). Changes in yields, essential oil and linalool contents of *Coriandrum sativum* varieties (var. *Vulgare* Alef. and var. *Microcarpum* DC.) harvested at different development stages. Eur. J. Hortic. Sci..

[71] Priyadarshi S., Borse B.B. (2014). Effect of the environment on content and composition of essential oil in coriander. Int. J. Sci. Eng. Res..

[72] Shams M., Esfahan S.Z., Esfahan E.Z., Dashtaki H.N., Dursun A., Yildirim E. (2016). Effects of climatic factors on the quantity of essential oil and dry matter yield of coriander (*Coriandrum sativum* L.). Indian J. Sci. Technol..

[73] Punetha D., Tewari G., Pande C. (2018). Compositional variability in inflorescence essential oil of *Coriandrum sativum* from North India. J. Essent. Oil Res..

[74] Gil A., De La Fuente E.B., Lenardis A.E., López Pereira M., Suárez S.A., Bandoni A., Van Baren C., Di Leo Lira P., Ghersa C.M. (2002). Coriander essential oil composition from two genotypes grown in different environmental conditions. J. Agric. Food Chem..

[75] İzgı M.N., Telci İ., Elmastaş M. (2017). Variation in essential oil composition of coriander (*Coriandrum sativum* L.) Varieties Cultivated in Two Different Ecologies. J. Essent. Oil Res..

[76] Msaada K., Taarit M.B., Hosni K., Hammami M., Marzouk B. (2009). Regional and maturational effects on essential oils yields and composition of coriander (*Coriandrum sativum* L.) fruits. Sci. Hortic..

[77] Gandova V., Tasheva S., Marinova K., Dimov M., Dobreva K., Prodanovastefanova V., Stoyanova A. (2020). Investigation of chemical composition, thermodynamic and thermal properties of coriander (*Coriandrum sativum*. L) essential oil. Oxid. Commun..

[78] Ravi R., Prakash M., Bhat K.K. (2007). Aroma Characterization of coriander (*Coriandrum sativum* L.) oil samples. Eur. Food Res. Technol..

[79] Al amrani K., Barbouchi M., Elidrissi M., Amechrouq A., Chokrad M. (2019). Chemical composition and physicochemical properties of the essential oil of coriander (*Coriandrum sativum* L.) grown in Morocco. RHAZES: Green App. Chem..

[80] Huzar E., Dzięcioł M., Wodnicka A., Örün H., İçöz A., Çiçek E. (2018). Influence of hydrodistillation conditions on yield and composition of coriander (*Coriandrum sativum* L.) essential oil. Pol. J. Food Nutr. Sci..

[81] Sumalan R.M., Alexa E., Popescu I., Negrea M., Radulov I., Obistioiu D., Cocan I. (2019). Exploring ecological alternatives for crop protection using *Coriandrum sativum* essential oil. Molecules.

[82] Choi S.-A., Lee H.-S. (2018). Insecticidal activities of Russia coriander oils and these constituents against *Sitophilus oryzae* and *Sitophilus zeamais*. J. Appl. Biol. Chem..

[83] Kiralan M., Calikoglu E., Ipek A., Bayrak A., Gurbuz B. (2009). Fatty Acid and volatile oil composition of different coriander (*Coriandrum sativum*) registered varieties cultivated in Turkey. Chem. Nat. Compo..

[84] Georgieva R., Delibaltova V., Chavdarov P. (2022). Change in agronomic characteristics and essential oil composition of coriander after application of foliar fertilizers and biostimulators. Ind. Crops Prod..

[85] Khalid K.A. (2015). Effect of macro and micro nutrients on essential oil of coriander fruits. J. Mat. Environ. Sci..

[86] Özyazici G. (2021). Influence of organic and inorganic fertilizers on coriander (*Coriandrum sativum* L.) agronomic traits, essential oil and components under semi-arid climate. Agronomy.

[87] Rasouli F., Nasiri Y., Asadi M., Hassanpouraghdam M.B., Golestaneh S., Pirsarandib Y. (2022). Fertilizer type and humic acid improve the growth responses, nutrient uptake, and essential oil content on *Coriandrum sativum* L.. Sci. Rep..

[88] Msaada K., Taârit M.B., Hosni K., Nidhal S., Tammar S., Bettaieb I., Hammami M., Limam F., Marzouk B. (2012). Comparison of different extraction methods for the determination of essential oils and related compounds from coriander (*Coriandrum sativum* L.). Acta Chim. Slov..

[89] Sourmaghi M.H.S., Kiaee G., Golfakhrabadi F., Jamalifar H., Khanavi M. (2015). Comparison of essential oil composition and antimicrobial activity of *Coriandrum sativum* L. extracted by hydrodistillation and microwave-assisted hydrodistillation. J. Food Sci. Technol..

[90] Reverchon E., De Marco I. (2006). Supercritical fluid extraction and fractionation of natural matter. J. Supercrit. Fluids.

[91] Pavlić B., Vidović S., Vladić J., Radosavljević R., Zeković Z. (2015). Isolation of coriander (*Coriandrum sativum* L.) essential oil by green extractions versus traditional techniques. J. Supercrit. Fluids.

[92] Song E.-J., Ko M.-J. (2022). Extraction of monoterpenes from coriander (*Coriandrum sativum* L.) Seeds Using Subcritical Water Extraction (SWE) Technique. J. Supercrit. Fluids.

[93] Nurzyńska-Wierdak R. (2013). Essential oil composition of the coriander (*Coriandrum sativum* L.) herb depending on the development stage. Acta Agrobot..

[94] Ghazanfari N., Mortazavi S.A., Yazdi F.T., Mohammadi M. (2020). Microwave-assisted hydrodistillation extraction of essential oil from coriander seeds and evaluation of their composition, antioxidant and antimicrobial activity. Heliyon.

[95] Palmieri S., Pellegrini M., Ricci A., Compagnone D., Lo Sterzo C. (2020). Chemical composition and antioxidant activity of thyme, hemp and coriander extracts: A comparison study of maceration, soxhlet, UAE and RSLDE techniques. Foods.

[96] Zekovic Z., Adamovic D., Cetkovic G., Radojkovic M., Vidovic S. (2011). Essential oil and extract of coriander (*Coriandrum sativum* L.). Acta Period. Technol..

[97] Ahmed Y.M., Sulaiman R.Z. (2018). Detection of some active compounds in the leaves and stems of local coriander plant-*Coriandrum sativum* L.. Tikrit J. Pure Sci..

[98] Darughe F., Barzegar M., Sahari M.A. (2012). Antioxidant and antifungal activity of coriander (*Coriandrum sativum* L.) essential oil in cake. Food Chem. Toxicol..

[99] Eikani M.H., Golmohammad F., Rowshanzamir S. (2007). Subcritical water extraction of essential oils from coriander seeds (*Coriandrum sativum* L.). J. Food Eng..

[100] Norashikin S., Rozita O., Wan A.H.M.Y., Rossuriati D.H. (2008). Subcritical water extraction of essential oil from coriander (*Coriandrum sativum* L.) Seeds. Malays. J. Anal. Sci..

[101] Mhemdi H., Rodier E., Kechaou N., Fages J. (2011). A supercritical tuneable process for the selective extraction of fats and essential oil from coriander seeds. J. Food Eng..

[102] Illés V., Daood H.G., Perneczki S., Szokonya L., Then M. (2000). Extraction of coriander seed oil by CO_2_ and propane at super- and subcritical conditions. J. Supercrit. Fluids.

[103] Anitescu G., Doneanu C., Radulescu V. (1997). Isolation of coriander oil: Comparison between steam distillation and supercritical CO_2_ extraction. Flavour Fragr. J..

[104] Shrirame B.S., Geed S.R., Raj A., Prasad S., Rai M.K., Singh A.K., Singh R.S., Rai B.N. (2018). Optimization of supercritical extraction of coriander (*coriandrum sativum* l.) seed and characterization of essential ingredients. J. Essent. Oil-Bear. Plants.

[105] Duarte A., Luís Â., Oleastro M., Domingues F.C. (2016). Antioxidant properties of coriander essential oil and linalool and their potential to control *Campylobacter* spp.. Food Control.

[106] Ramadan M.F., Kroh L.W., Mörsel J.-T. (2003). Radical scavenging activity of black cumin (*Nigella sativa* L.), coriander (*Coriandrum sativum* L.), and niger (*Guizotia abyssinica* cass.) crude seed oils and oil fractions. J. Agric. Food Chem..

[107] Guerra N.B., de Almeida Melo E., Filho J.M. (2005). antioxidant compounds from coriander (*Coriandrum sativum* L.) etheric extract. J. Food Compost. Anal..

[108] Önder A., El-Shemy H.A. (2018). Coriander and its phytoconstituents for the beneficial effects. Potential of Essential Oils.

[109] Baccouri B., Rajhi I., Perveen S., Al-Taweel A.M. (2021). Potential antioxidant activity of terpenes. Terpenes and Terpenoids-Recent Advances.

[110] Dua A., Agrawal S., Kaur A., Mahajan R. (2014). Antioxidant profile of *Coriandrum sativum* methanolic extract. Int. Res. J. Pharm..

[111] Ani V., Varadaraj M.C., Naidu K.A. (2006). Antioxidant and antibacterial activities of polyphenolic compounds from bitter cumin (*Cuminum nigrum* L.). Eur. Food Res. Technol..

[112] Raghuramulu N., Madhavan Nair K., Kalyanasundaram S. (1983). A Manual of Laboratory Techniques.

[113] Dua A., Mittal A., Gupta S., Mahajan R. (2013). Bioreactive compounds and antioxidant properties of methanolic extract of fennel (*Foeniculum vulgare* Miller). Int. Res. J. Pharm..

[114] Slinkard K., Singleton V.L. (1977). Total phenol analysis: Automation and comparison with manual methods. Am. J. Enol. Vitic..

[115] Mehdizadeh L., Moghaddam M., Grumezescu A.M., Holban A.M. (2018). Essential oils: Biological activity and therapeutic potential. Therapeutic, Probiotic, and Unconventional Foods.

[116] Delaquis P.J., Stanich K., Girard B., Mazza G. (2002). Antimicrobial activity of individual and mixed fractions of dill, cilantro, coriander and eucalyptus essential oils. Int. J. Food Microbiol..

[117] Keskin D., Toroglu S. (2011). Studies on antimicrobial activities of solvent extracts of different spices. J. Environ. Biol..

[118] Duarte A., Ferreira S., Silva F., Domingues F.C. (2012). Synergistic activity of coriander oil and conventional antibiotics against *Acinetobacter baumannii*. Phytomedicine.

[119] Zare-Shehneh M., Askarfarashah M., Ebrahimi L., Kor N.M., Zare-Zardini H., Soltaninejad H., Hashemian Z., Jabinian F. (2014). Biological activities of a new antimicrobial peptide from *Coriandrum sativum*. Int. J. Biosci..

[120] Silva F., Domingues F.C. (2017). Antimicrobial activity of coriander oil and its effectiveness as food preservative. Crit. Rev. Food Sci. Nutr..

[121] Innocent B.X. (2011). Studies on the immunostimulant activity of *Coriandrum sativum* and resistance to *Aeromonas hydrophila* in *Catla catla*. J. Appl. Pharm. Sci..

[122] Kačániová M., Galovičová L., Ivanišová E., Vukovic N.L., Štefániková J., Valková V., Borotová P., Žiarovská J., Terentjeva M., Felšöciová S. (2020). Antioxidant, antimicrobial and antibiofilm activity of coriander (*Coriandrum sativum* L.) Essential Oil for Its Application in Foods. Foods.

[123] Bajpai V.K., Sharma A., Baek K.-H. (2013). Antibacterial mode of action of *Cudrania tricuspidata* fruit essential oil, affecting membrane permeability and surface characteristics of food-borne pathogens. Food Control.

[124] de Freires I.A., Murata R.M., Furletti V.F., Sartoratto A., de Alencar S.M., Figueira G.M., de Oliveira Rodrigues J.A., Duarte M.C.T., Rosalen P.L. (2014). *Coriandrum sativum* L. (coriander) essential oil: Antifungal activity and mode of action on *Candida* spp., and molecular targets affected in human whole-genome expression. PLoS ONE.

[125] Lalitha V., Kiran B., Raveesha K.A. (2011). Antifungal and antibacterial potentiality of six essential oils extracted from plant source. Int. J. Eng. Sci. Technol..

[126] Soares B.V., Morais S.M., dos Santos Fontenelle R.O., Queiroz V.A., Vila-Nova N.S., Pereira C.M.C., Brito E.S., Neto M.A.S., Brito E.H.S., Cavalcante C.S.P. (2012). Antifungal activity, toxicity and chemical composition of the essential oil of *Coriandrum sativum* L. Fruits. Molecules.

[127] Helal M.A., Abdel-Gawad A.M., Kandil O.M., Khalifa M.M.E., Cave G.W.V., Morrison A.A., Bartley D.J., Elsheikha H.M. (2020). Nematocidal effects of a coriander essential oil and five pure principles on the infective larvae of major ovine gastrointestinal nematodes in vitro. Pathogens.

[128] Eguale T., Tilahun G., Debella A., Feleke A., Makonnen E. (2007). In vitro and in vivo anthelmintic activity of crude extracts of *Coriandrum sativum* against *Haemonchus contortus*. J. Ethnopharmacol..

[129] Zoubiri S., Baaliouamer A. (2010). Essential oil composition of *Coriandrum sativum* seed cultivated in Algeria as food grains protectant. Food Chem..

[130] Ngamo T., Ngatanko I., Ngassou M., Mapongmestem P., Hance T. (2007). Insecticidal efficiency of essential oils of 5 aromatic plants tested both alone and in combination towards *Sitophilus oryzae* (L.) (Coleoptera: Curculionidae). J. Adv. Pharm. Technol. Res..

[131] Stejskal V., Vendl T., Aulicky R., Athanassiou C. (2021). Synthetic and natural insecticides: Gas, liquid, gel and solid formulations for stored-product and food-industry pest control. Insects.

[132] Hansen L.S., Jensen K.M.V. (2002). Effect of temperature on parasitism and host-Feeding of *Trichogramma turkestanica* (Hymenoptera: Trichogrammatidae) on *Ephestia kuehniella* (Lepidoptera: Pyralidae). J. Econ. Entomol..

[133] Ayvaz A., Albayrak S., Karaborklu S. (2008). Gamma radiation sensitivity of the eggs, larvae and pupae of Indian meal moth *Plodia interpunctella* (Hübner) (Lepidoptera: Pyralidae). Pest Manag. Sci..

[134] Sighamony S., Anees I., Chandrakala T., Osmani Z. (1986). Efficacy of certain indigenous plant products as grain protectants against *Sitophilus oryzae* (L.) and *Rhyzopertha dominica* (F.). J. Stored Prod. Res..

[135] SritiEljazi J., Bachrouch O., Salem N., Msaada K., Aouini J., Hammami M., Boushih E., Abderraba M., Limam F., Mediouni Ben Jemaa J. (2017). Chemical composition and insecticidal activity of essential oil from coriander fruit against *Tribolium castaenum*, *Sitophilus oryzae*, and *Lasioderma serricorne*. Int. J. Food Prop..

[136] López M.D., Jordán M.J., Pascual-Villalobos M.J. (2008). Toxic compounds in essential oils of coriander, caraway and basil active against stored rice pests. J. Stored Prod. Res..

[137] Islam M.S., Hasan M.M., Xiong W., Zhang S.C., Lei C.L. (2009). Fumigant and repellent activities of essential oil from *Coriandrum sativum* (L.) (Apiaceae) against red flour beetle *Tribolium castaneum* (Herbst) (Coleoptera: Tenebrionidae). J. Pest Sci..

[138] Khani A., Rahdari T. (2012). Chemical composition and insecticidal activity of essential oil from *Coriandrum sativum* seeds against *Tribolium confusum* and *Callosobruchus maculatus*. ISRN Pharm..

[139] Aligita W., Susilawati E., Septiani H., Atsil R. (2018). Antidiabetic activity of Coriander (*Coriandrum sativum* L.) leaves’ ethanolic extract. Int. J. Pharm. Biol. Arch..

[140] Lipinski B. (2001). Pathophysiology of oxidative stress in diabetes mellitus. J. Diabetes Complicat..

[141] Dakhlaoui S., Wannes W.A., Sari H., Hmida M.B., Frouja O., Limam H., Tammar S., Bachkouel S., Jemaa M.B., Jallouli S. (2022). Combined effect of essential oils from Lavender (*Lavandula officinalis* L.) aerial parts and coriander (*Coriandrum sativum* L.) seeds on antioxidant, anti-diabetic, anti-cancer and anti-inflammatory activities. J. Essent. Oil-Bear. Plants..

[142] Swanston-Flatt S.K., Day C., Bailey C.J., Flatt P.R. (1990). Traditional plant treatments for diabetes. Studies in normal and streptozotocin diabetic mice. Diabetologia.

[143] Mahmoud M.F., Ali N., Mostafa I., Hasan R.A., Sobeh M. (2022). Coriander oil reverses dexamethasone-induced insulin resistance in rats. Antioxidants.

[144] El-Soud N.H.A., El-Lithy N.A., El-Saeed G.S.M., Wahby M.S., Khalil M.Y., El-Kassem L.T.A., Morsy F., Shaffie N. (2012). Efficacy of *Coriandrum sativum* L. essential oil as antidiabetic. J. Appl. Sci. Res..

[145] Deepa B., Venkatraman Anuradha C. (2013). Effects of linalool on inflammation, matrix accumulation and podocyte loss in kidney of streptozotocin-induced diabetic rats. Toxicol. Mech. Methods.

[146] More T.A., Kulkarni B.R., Nalawade M.L., Arvindekar A.U. (2014). Antidiabetic activity of linalool and limonene in streptozotocin-induced diabetic rat: A combinatorial therapy approach. Int. J. Pharm. Pharm. Sci..

[147] Garikiparithi M. 10 Best Essential Oils for High Cholesterol Reduction. https://www.belmarrahealth.com/10-best-essential-oils-high-cholesterol-reduction/.

[148] Lal A.A.S., Kumar T., Murthy P.B., Pillai K.S. (2004). Hypolipidemic effect of *Coriandrum sativum* L. in triton-induced hyperlipidemic rats. Indian J. Exp. Biol..

[149] Ramadan M.F., Amer M.M.A., Awad A.E.-S. (2008). Coriander (*Coriandrum sativum* L.) seed oil improves plasma lipid profile in rats fed a diet containing cholesterol. Eur. Food Res. Technol..

[150] Vimala G., Gricilda Shoba F. (2014). A review on antiulcer activity of few Indian medicinal plants. Int. J. Microbiol..

[151] de Oliveira F.A., Andrade L.N., de Sousa E.B.V., de Sousa D.P. (2014). Anti-ulcer activity of essential oil constituents. Molecules.

[152] Heidari B., Sajjadi S.E., Minaiyan M. (2016). Effect of *Coriandrum sativum* hydroalcoholic extract and its essential oil on acetic acid- induced acute colitis in rats. Avicenna J. Phytomed..

[153] Jia X.-Y., Zhang Q.-A., Zhang Z.-Q., Wang Y., Yuan J.-F., Wang H.-Y., Zhao D. (2011). Hepatoprotective effects of almond oil against carbon tetrachloride induced liver injury in rats. Food Chem..

[154] Ben Hsouna A., Dhibi S., Dhifi W., Mnif W., Ben Nasr H., Hfaiedh N. (2019). Chemical composition and Hepatoprotective effect of essential oil from *Myrtus communis* L. flowers against CCL4-induced acute hepatotoxicity in rats. RSC Adv..

[155] Cardia G.F.E., de Souza Silva-Comar F.M., Silva E.L., da Rocha E.M.T., Comar J.F., Silva-Filho S.E., Zagotto M., Uchida N.S., Bersani-Amado C.A., Cuman R.K.N. (2021). Lavender (*Lavandula officinalis*) essential oil prevents acetaminophen-induced hepatotoxicity by decreasing oxidative stress and inflammatory response. Res. Soc. Dev..

[156] Özbek H., Kırmızı N.İ., Cengiz N., Erdoğan E. (2016). Hepatoprotective effects of *Coriandrum sativum* essential oil against acute hepatotoxicity induced by carbon tetrachloride on rats. ACTA Pharm. Sci..

[157] Altınok-Yipel F., Ozan Tekeli İ., Özsoy Ş.Y., Güvenç M., Kaya A., Yipel M. (2020). Hepatoprotective activity of linalool in rats against liver injury induced by carbon tetrachloride. Int. J. Vitam. Nutr. Res..

[158] Mazani M., Rezagholizadeh L., Shamsi S., Mahdavifard S., Ojarudi M., Salimnejad R., Salimi A. (2022). protection of CCl4-induced hepatic and renal damage by linalool. Drug Chem. Toxicol..

[159] Hsouna A.B., Sadaka C., Beyrouthy M.E., Hfaiedh M., Dhifi W., Brini F., Saad R.B., Mnif W. (2022). Immunomodulatory effect of linalool (Lin) against CCl_4_ -induced hepatotoxicity and oxidative damage in rats. Biotechnol. Appl. Biochem..

[160] Scattergood G. Apiaceous Opportunity: Coriander Oil Displays Anti-Ageing Skin Care Nenefits—New Research. https://www.cosmeticsdesign-asia.com/Article/2022/05/04/corinader-oil-has-the-potential-to-be-effective-anti-ageing-ingredient.

[161] Salem M.A., Manaa E.G., Osama N., Aborehab N.M., Ragab M.F., Haggag Y.A., Ibrahim M.T., Hamdan D.I. (2022). Coriander (*Coriandrum sativum* L.) essential oil and oil-loaded nano-formulations as an anti-aging potentiality via TGFβ/SMAD pathway. Sci. Rep..

[162] Bahr T.A., Rodriguez D., Beaumont C., Allred K. (2019). The effects of various essential oils on epilepsy and acute seizure: A systematic review. Evid. Based. Complement. Alternat. Med..

[163] Gastón M.S., Cid M.P., Vázquez A.M., Decarlini M.F., Demmel G.I., Rossi L.I., Aimar M.L., Salvatierra N.A. (2016). Sedative effect of central administration of *Coriandrum sativum* essential oil and its major component linalool in neonatal chicks. Pharm. Biol..

[164] Emam G.M., Heydari H.G. (2008). Effect of extract and essential oil of *Coriandrum sativum* seed against Pentylenetetrazole induced seizure. Pharm. Sci..

[165] Olivares D., Deshpande V.K., Shi Y., Lahiri D.K., Greig N.H., Rogers J.T., Huang X. (2012). N-Methyl D-Aspartate (NMDA) receptor antagonists and memantine treatment for Alzheimer’s disease, vascular dementia and Parkinson’s disease. Curr. Alzheimer Res..

[166] NIMH Anxiety Disorders. https://www.nimh.nih.gov/health/topics/anxiety-disorders.

[167] Setzer W.N. (2009). Essential oils and anxiolytic aromatherapy. Nat. Prod. Commun..

[168] Cioanca O., Hritcu L., Mihasan M., Trifan A., Hancianu M. (2014). Inhalation of coriander volatile oil increased anxiolytic-antidepressant-like behaviors and decreased oxidative status in beta-amyloid (1-42) Rat model of Alzheimer’s disease. Physiol. Behav..

[169] Oerke E.-C. (2006). Crop Losses to Pests. J. Agric. Sci..

[170] Murphy K.M., Dawson J.C., Jones S.S. (2008). Relationship among phenotypic growth traits, yield and weed suppression in spring wheat landraces and modern cultivars. Field Crops Res..

[171] Kraehmer H., Laber B., Rosinger C., Schulz A. (2014). Herbicides as weed control agents: State of the art: I. Weed Control Research and Safener Technology: The Path to Modern Agriculture. Plant Physiol..

[172] Kaur S., Singh H.P., Batish D.R., Kohli R.K. (2011). Chemical characterization and allelopathic potential of volatile oil of *Eucalyptus tereticornis* against *Amaranthus viridis*. J. Plant Interact..

[173] Dayan F.E., Cantrell C.L., Duke S.O. (2009). Natural products in crop protection. Bioorg. Med. Chem..

[174] Azirak S., Karaman S. (2008). Allelopathic effect of some essential oils and components on germination of weed species. Acta Agric. Scand. Sect. B Soil Plant Sci..

[175] Dhima K., Vasilakoglou I., Garane V., Ritzoulis C., Lianopoulou V., Panou-Philotheou E. (2010). Competitiveness and essential oil phytotoxicity of seven annual aromatic plants. Weed Sci..

[176] Rahimi A.R., Mousavizadeh S.J., Mohammadi H., Rokhzadi A., Majidi M., Amini S. (2013). Allelopathic effect of some essential oils on seed germination of *Lathyrus annuus* and *Vicia villosa*. J. Biodivers..

